# Association of three promoter polymorphisms in interleukin-10 gene with cancer susceptibility in the Chinese population: a meta-analysis

**DOI:** 10.18632/oncotarget.18220

**Published:** 2017-05-26

**Authors:** Ping Wang, Junling An, Yanfeng Zhu, Xuedong Wan, Hongzhen Zhang, Shoumin Xi, Sanqiang Li

**Affiliations:** ^1^ The Key Laboratory of Pharmacology and Medical Molecular Biology, Medical College, Henan University of Science and Technology, Luoyang 471023, Henan, China; ^2^ The Molecular Medicine Key Laboratory of Liver Injury and Repair, Medical College, Henan University of Science and Technology, Luoyang 471023, Henan, China

**Keywords:** interleukin-10, polymorphism, cancer, susceptibility, meta-analysis

## Abstract

Numerous studies have examined the associations of three promoter polymorphisms (-1082A/G, -819T/C and -592A/C) in *IL-10* gene with cancer susceptibility in the Chinese population, but the results remain inconclusive. To gain a more precise estimation of this potential association, we conducted the current meta-analysis based on 53 articles, including 26 studies with 4,901 cases and 6,426 controls for the -1082A/G polymorphism, 33 studies with 6,717 cases and 8,550 controls for the -819T/C polymorphism, and 42 studies with 9,934 cases and 13,169 controls for the -592A/C polymorphism. Pooled results indicated that the three promoter polymorphisms in *IL-10* gene were significantly associated with an increased overall cancer risk in the Chinese population. Stratification analysis showed that the association was more pronounced for hepatocellular carcinoma and low quality studies for the -1082A/G polymorphism, lung cancer and oral cancer for the -819T/C polymorphism. However, the -592A/C polymorphism was associated with a statistically significant increased risk for lung cancer, oral cancer, hospital-based studies and low quality studies, but a decreased risk for colorectal cancer. We further investigated the significant results using the false-positive report probability (FPRP) test. Interestingly, FPRP test results revealed that only *IL-10* -1082A/G polymorphism was truly associated with an increased overall cancer risk. In the subgroup analysis, only the low quality studies, lung cancer and colorectal cancer remained significant at the prior level of 0.1. Although this association needs further confirmation by considering large studies, this meta-analysis suggested an association between *IL-10* gene polymorphisms and cancer risk in the Chinese population.

## INTRODUCTION

Cancer is still a global public health problem. According to the GLOBOCAN estimates, about 14.1 million new cancer cases and 8.2 million deaths occurred in 2012 worldwide [1]. In China, cancer has become the leading cause of death since 2010, with an estimate of 4292,000 new cancer cases and 2814,000 cancer deaths in 2015 [2]. As a multifactorial disease, it involves both genetic and environmental factors [3]. Accumulating evidence has indicated that inflammation plays a vital role in cancer development [4–6], and approximately 20% of all cancers are associated with chronic inflammation [7].

Interleukin-10 (IL-10) is an anti-inflammatory cytokine with both immunosuppressive and immunostimulatory activities [8]. Although the relationship between IL-10 and cancer has been extensively studied, the exact role of IL-10 in cancer is still elusive, since IL-10 have both cancer-promoting and -inhibiting properties [9, 10]. In view of these properties, we hypothesized that *IL-10* gene polymorphisms could influence cancer susceptibility.

The *IL-10* gene is located on chromosome 1q31-32, and is composed of five exons and four introns. *IL-10* gene promoter region is highly polymorphic, and three promoter single nucleotide polymorphisms (SNPs) such as -1082A/G (rs1800896), -819T/C (rs1800871) and -592A/C (rs1800872) have been reported to regulate IL-10 expression [11, 12] and alter the susceptibility to various types of cancers [13–16]. In the Chinese population, numerous case-control studies were performed to investigate the role of *IL-10* -1082A/G, -819T/C and -592A/C polymorphisms in cancer risk. However, the results remain inconclusive. Hence, we performed the present meta-analysis to investigate the association between three polymorphisms in *IL-10* gene and cancer susceptibility in the Chinese population.

## RESULTS

### Study characteristics

**Figure 1:**
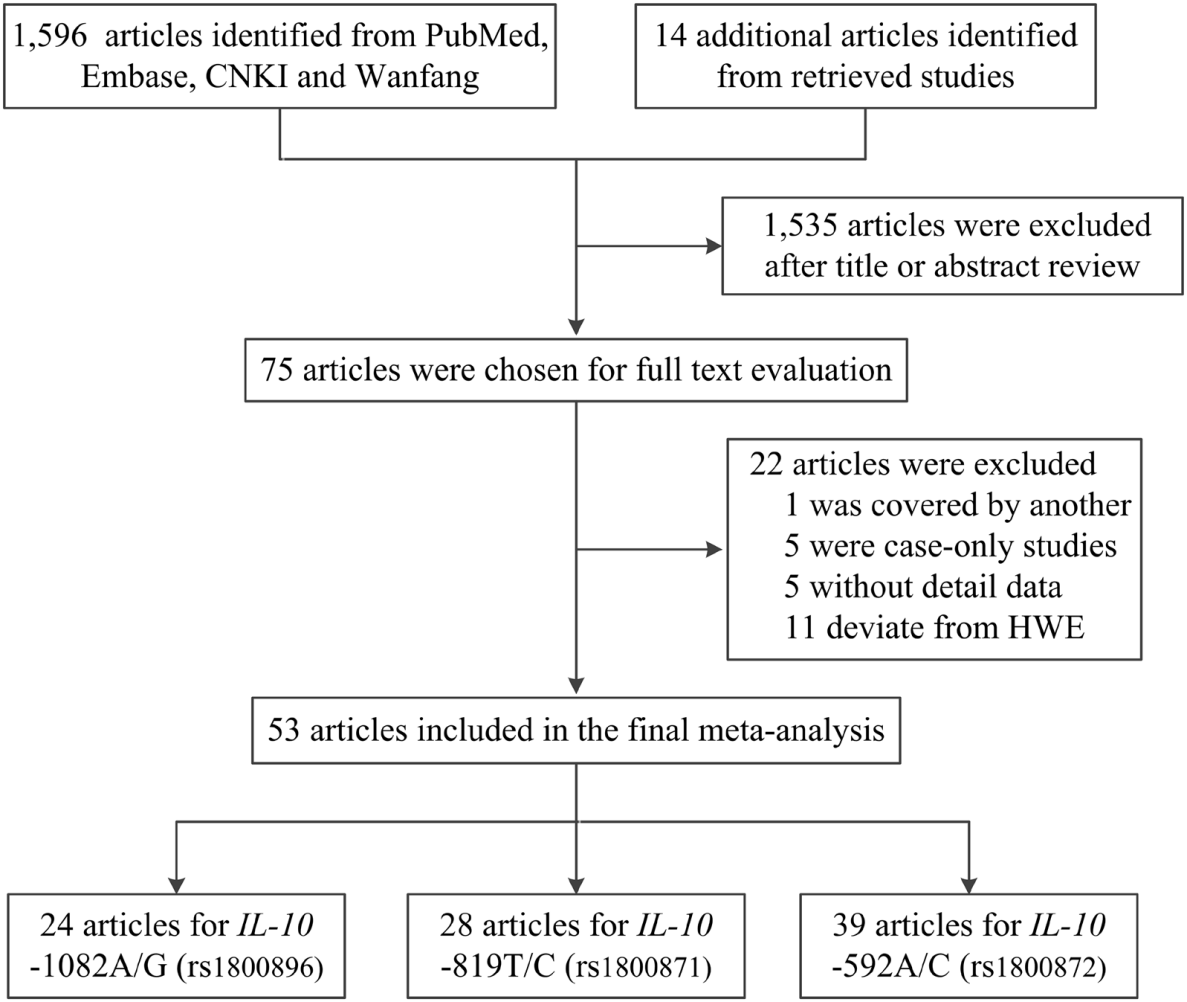
Flow diagram of the study selection process

As shown in Figure 1, 1,596 published records were initially retrieved from PubMed, Embase, Chinese National Knowledge Infrastructure (CNKI) and Wanfang database, and 14 more articles were identified by checking the references in the retrieved publications. After reviewing of the titles and abstracts, 1,535 articles were excluded, leaving only 75 articles for further assessment. Among them, we excluded one study [17] that was covered by another included publication [18], five case-only studies [19–23], five lacking detailed data for further analysis [24–28], and eleven that were considering the deviation from the Hardy-Weinberg equilibrium (HWE) [29–39]. Ultimately, 53 articles were included in the final meta-analysis. Of these 53 articles, 24 articles [40–63] include 26 studies examining *IL-10* -1082A/G polymorphism, 28 articles [18, 42, 43, 45, 47, 49, 52, 53, 57-61, 63-77] include 33 studies examining the -819T/C polymorphism, and 39 articles [18, 42, 43, 45, 47, 52, 53, 56-67, 69, 70, 73-76, 78-91] include 42 studies examining the -592A/C polymorphism (Table 1). Of the 53 articles, two publications [18, 45] with three cancer types were considered as three studies and one publication [65] with two cancer types were also considered as two studies.

**Table 1:** Characteristics of studies included in the meta-analysis

Surname [ref]	Year	Cancer type	Control source	Genotype method	Case				Control				MAF	HWE	Score
					11	12	22	All	11	12	22	All			
**-1082A/G polymorphism**															
Wu [40]	2002	Gastric	HB	Sequencing	135	14	1	150	208	11	1	220	0.03	0.057	6
Heneghan [41] ^a^	2003	HCC	PB	Probe	86	12	0	98	90	7	0	97	0.04	0.712	10
Shih [42]^a^	2005	Lung	HB	PCR-RFLP	115	39	0	154	194	11	0	205	0.03	0.693	8
Wei [43]	2007	NPC	HB	PCR-RFLP	123	61	14	198	167	38	5	210	0.11	0.124	8
Bai [44]^b^	2008	Gastric	HB	PCR-RFLP	89	22 (AG+GG)		111	104	7 (AG+GG)		111	NA	NA	7
Hsing [45]	2008	Gallbladder	PB	Taqman	231	23	1	255	624	99	7	730	0.08	0.173	12
Hsing [45] ^a^	2008	EHBD	PB	Taqman	107	18	0	125	664	108	7	779	0.08	0.270	12
Hsing [45]^a^	2008	AV	PB	Taqman	38	9	0	47	664	108	7	779	0.08	0.270	12
Hao [46] ^b^	2009	Lung	PB	Taqman	36	7 (AG+GG)		43	46	6 (AG+GG)		52	NA	NA	7
Xiao [47] ^a^	2009	Gastric	HB	PCR-RFLP	176	41	3	220	593	31	0	624	0.03	0.525	9
Kong [48]	2010	Breast	HB	PCR-RFLP	285	29	1	315	285	35	2	322	0.06	0.422	9
Liu [49]	2010	HCC	HB	Taqman	131	35	4	170	160	24	3	187	0.08	0.075	5
Niu [50] ^b^	2011	Prostate	PB	Sequencing	24	74 (AG+GG)		98	42	46 (AG+GG)		88	NA	NA	9
Wang [51]	2011	Cervical	PB	PCR-SSP	77	85	24	186	103	76	21	200	0.30	0.222	8
He [52] ^a^	2012	Gastric	HB	PCR-RFLP	154	42	0	196	194	54	0	248	0.11	0.055	9
Chang [53] ^a^	2013	HN	HB	Taqman	289	23	1	313	268	27	0	295	0.05	0.410	10
Chen [54]	2013	Bladder	HB	AS-PCR	374	25	1	400	350	48	2	400	0.07	0.799	10
Du [55]	2013	Esophageal	HB	PCR	95	20	3	118	103	15	1	119	0.07	0.587	8
Pan [56]	2013	Gastric	HB	MassARRAY	263	41	4	308	264	41	3	308	0.08	0.329	9
Cheng [57] ^a^	2015	NTCL	HB	PCR-LDR	101	24	0	125	237	60	3	300	0.11	0.710	10
Fei [58]	2015	AML	HB	PCR-RFLP	75	70	22	167	159	134	35	328	0.31	0.398	8
Hsu [59] ^a^	2015	Oral	HB	PCR-SSP	130	14	1	145	96	16	0	112	0.07	0.416	7
Yang [60]	2015	Esophageal	HB	MassARRAY	41	106	99	246	46	204	242	492	0.30	0.751	9
Bai [61]	2016	Cervical	HB	PCR-RFLP	74	75	16	165	80	72	13	165	0.30	0.563	8
Cai [62] ^a^	2016	Colorectal	HB	MassARRAY	323	50	2	375	343	39	0	382	0.05	0.293	9
Peng [63]	2016	HCC	PB	PCR-RFLP	83	74	16	173	96	74	12	182	0.27	0.653	10
**-819T/C polymorphism**															
Wu [64]	2003	Gastric	HB	Sequencing	88	105	27	220	127	83	20	230	0.27	0.231	9
Savage [65]	2004	Gastric	PB	SBE	37	38	9	84	170	163	49	382	0.34	0.315	11
Savage [65]	2004	Esophageal	PB	SBE	53	46	17	116	170	163	49	382	0.34	0.315	12
Shih [42]	2005	Lung	HB	PCR-RFLP	66	58	30	154	104	86	15	205	0.28	0.627	8
Wei [43]	2007	NPC	HB	PCR-RFLP	82	81	35	198	94	92	24	210	0.33	0.836	8
Hsing [45]	2008	Gallbladder	PB	Taqman	122	92	23	237	311	335	82	728	0.34	0.564	12
Hsing [45]	2008	EHBD	PB	Taqman	55	52	17	124	334	353	90	777	0.34	0.823	12
Hsing [45]	2008	AV	PB	Taqman	20	6	21	47	334	353	90	777	0.34	0.823	12
Yao [66]	2008	Oral	HB	PCR-RFLP	113	120	47	280	129	134	37	300	0.35	0.809	10
Xiao [47]	2009	Gastric	HB	PCR-RFLP	100	100	20	220	272	283	69	624	0.34	0.719	9
Liu [67]	2010	Prostate	HB	PCR-RFLP	120	108	34	262	132	110	28	270	0.31	0.477	10
Liu [49]	2010	HCC	HB	Taqman	79	73	18	170	75	92	20	187	0.35	0.292	5
Oh [18]	2010	Esophageal	PB	Taqman	90	79	27	196	179	158	42	379	0.32	0.426	13
Oh [18]	2010	Gastric	PB	Taqman	81	87	20	188	179	158	42	379	0.32	0.426	13
Oh [18]	2010	HCC	PB	Taqman	91	70	25	186	179	158	42	379	0.32	0.426	13
Su [68]	2010	Gastric	HB	PCR-RFLP	18	21	4	43	51	43	6	100	0.28	0.433	6
Bei [69]	2011	HCC	HB	Taqman	44	247	298	589	51	240	306	597	0.29	0.686	12
Liu [70]	2011	Gastric	HB	PCR-RFLP	99	96	39	234	109	106	28	243	0.33	0.773	7
He [52]	2012	Gastric	HB	PCR-RFLP	82	96	18	196	92	128	28	248	0.37	0.095	9
He [71]	2012	Breast	HB	MALDI-TOF MS	177	141	29	347	229	223	44	496	0.31	0.322	10
Yuan [72]	2012	Gastric	HB	MassARRAY	108	129	42	279	142	120	34	296	0.32	0.266	9
Zeng [73]	2012	Gastric	PB	SBE	60	80	11	151	78	65	10	153	0.28	0.467	10
Chang [53]	2013	HN	HB	Taqman	132	153	28	313	136	130	29	295	0.32	0.798	10
Yao [74]	2013	AML	HB	PCR-RFLP	68	38	9	115	56	63	18	137	0.36	0.966	9
Cheng [57]	2015	NTCL	HB	PCR-LDR	57	59	9	125	136	125	39	300	0.34	0.230	10
Fei [58]	2015	AML	HB	PCR-RFLP	57	72	38	167	137	137	54	328	0.37	0.052	8
Hsu [59]	2015	Oral	HB	PCR-SSP	33	101	11	145	53	51	8	112	0.30	0.363	7
Yang [60]	2015	Esophageal	HB	MassARRAY	101	105	40	246	219	203	69	492	0.35	0.051	9
Zhang [75]	2015	Lung	HB	PCR-RFLP	108	135	87	330	145	144	47	336	0.35	0.247	8
Bai [61]	2016	Cervical	HB	PCR-RFLP	44	76	45	165	28	73	64	165	0.39	0.362	8
Cui [76]	2016	Osteosarcoma	HB	PCR-RFLP	34	120	106	260	43	118	99	260	0.39	0.438	10
Li [77]	2016	Gastric	HB	PCR-RFLP	36	83	38	157	36	127	85	248	0.40	0.300	6
Peng [63]	2016	HCC	PB	PCR-RFLP	74	77	22	173	86	78	17	181	0.31	0.910	10
**-592A/C polymorphism**															
Wu [64]	2003	Gastric	HB	Sequencing	88	105	27	220	127	83	20	230	0.27	0.231	9
Savage [65]	2004	Gastric	PB	SBE	9	39	36	84	49	166	171	386	0.34	0.383	11
Savage [65]	2004	Esophageal	PB	SBE	17	51	51	119	49	166	171	386	0.34	0.383	12
Shih [42]	2005	Lung	HB	PCR-RFLP	66	70	18	154	116	76	13	205	0.25	0.907	8
Tseng [78]	2006	HCC	HB	MALDI-TOF MS	93	84	31	208	90	75	19	184	0.31	0.567	7
Wei [43]	2007	NPC	HB	PCR-RFLP	82	81	35	198	94	92	24	210	0.33	0.836	8
Hsing [45]	2008	Gallbladder	PB	Taqman	121	91	23	235	318	334	82	734	0.34	0.684	12
Yao [66]	2008	Oral	HB	PCR-RFLP	113	120	47	280	129	134	37	300	0.35	0.809	10
Xiao [47]	2009	Gastric	HB	PCR-RFLP	100	100	20	220	272	283	69	624	0.34	0.719	9
Liu [67]	2010	Prostate	HB	PCR-RFLP	120	108	34	262	132	110	28	270	0.31	0.477	10
Oh [18]	2010	Esophageal	PB	SNPlex	81	72	26	179	167	159	36	362	0.32	0.837	13
Oh [18]	2010	Gastric	PB	SNPlex	77	81	20	178	167	159	36	362	0.32	0.837	13
Oh [18]	2010	HCC	PB	SNPlex	82	68	19	169	167	159	36	362	0.32	0.837	13
Xiong [79]	2010	Cervical	HB	PCR-RFLP	35	23	12	70	51	44	13	108	0.32	0.467	7
Bei [69]	2011	HCC	HB	Taqman	42	248	299	589	49	244	304	597	0.29	0.997	12
Liang [80]	2011	Lung	HB	PCR-RFLP	69	36	11	116	69	44	7	120	0.24	0.997	9
Liu [70]	2011	Gastric	HB	PCR-RFLP	99	96	39	234	109	106	28	243	0.33	0.773	7
Yu [81]	2011	Cervical	PB	PCR-RFLP	59	37	7	103	52	44	19	115	0.36	0.075	10
He [52]	2012	Gastric	HB	PCR-RFLP	82	96	18	196	92	128	28	248	0.37	0.095	9
Zeng [73]	2012	Gastric	PB	SBE	59	77	15	151	80	66	7	153	0.26	0.148	10
Zhang [82]	2012	NHL	PB	Taqman	226	228	60	514	269	235	53	557	0.31	0.872	14
Chang [53]	2013	HN	HB	Taqman	134	152	27	313	137	129	29	295	0.32	0.864	10
Pan [56]	2013	Gastric	HB	MassARRAY	144	128	36	308	142	135	31	308	0.32	0.896	9
Sun [83]	2013	Esophageal	HB	SNPscan	162	163	31	356	191	141	33	365	0.28	0.347	10
Tsai [84]	2013	NPC	HB	PCR-RFLP	93	66	17	176	261	205	56	522	0.30	0.103	9
Yao [74]	2013	AML	HB	PCR-RFLP	68	38	9	115	56	63	18	137	0.36	0.966	9
Bei [85]	2014	HCC	HB	Taqman	356	312	52	720	392	313	79	784	0.30	0.160	11
Hsia [86]	2014	Lung	HB	PCR-RFLP	173	145	40	358	368	277	71	716	0.29	0.080	12
Kuo [87]	2014	Gastric	HB	PCR-RFLP	186	134	38	358	180	141	37	358	0.30	0.235	9
Yu [88]	2014	Colorectal	PB	PCR-RFLP	153	114	31	298	118	135	38	291	0.36	0.950	13
Cheng [57]	2015	NTCL	HB	PCR-LDR	57	59	9	125	138	124	38	300	0.33	0.225	10
Fei [58]	2015	AML	HB	PCR-RFLP	54	74	39	167	126	142	59	328	0.40	0.091	8
Hsu [59]	2015	Oral	HB	PCR-SSP	33	101	11	145	53	51	8	112	0.30	0.363	7
Yang [60]	2015	Esophageal	HB	MassARRAY	85	116	45	246	185	228	79	492	0.39	0.534	9
Yin [89]	2015	Gastric	HB	SNPscan	112	96	20	228	235	184	42	461	0.29	0.491	10
Zhang [75]	2015	Lung	HB	PCR-RFLP	64	156	110	330	85	176	75	336	0.49	0.374	8
Bai [61]	2016	Cervical	HB	PCR-RFLP	63	82	20	165	70	80	15	165	0.33	0.243	8
Cai [62]	2016	Colorectal	HB	MassARRAY	221	128	26	375	184	158	40	382	0.31	0.485	9
Chang [90]	2016	RCC	HB	PCR-RFLP	61	27	4	92	371	185	24	580	0.20	0.877	9
Cui [76]	2016	Osteosarcoma	HB	PCR-RFLP	108	125	27	260	100	128	32	260	0.37	0.359	10
Peng [63]	2016	HCC	PB	PCR-RFLP	57	81	35	173	79	81	22	182	0.34	0.860	10
Ma [91]	2016	Gastric	HB	PCR-RFLP	67	63	17	147	71	67	12	150	0.30	0.486	8

MAF: minor allele frequency; HWE: Hardy-Weinberg equilibrium; HB: hospital based; PB: population based; NA: not applicable; HCC: hepatocellular carcinoma; NPC: nasopharyngeal carcinoma; EHBD: extrahepatic bile duct; AV: ampulla of vater; HN: head and neck; NTCL: NK/T-cell lymphoma; AML: acute myeloid leukemia; NHL: non-Hodgkin’s lymphoma; RCC: renal cell carcinoma; PCR-RFLP: polymorphism chain reaction restriction fragment length polymorphism; PCR-SSP: polymerase chain reaction sequence-specific primer; AS-PCR: allele-specific polymorphism chain reaction; PCR-LDR: polymorphism chain reaction-ligase detection reaction; SBE: single base extension; MALDI-TOF MS: matrix-assisted laser desorption/ionization-time-of-flight mass spectrometry.

^a^ Heneghan [41], Shih [42], Hsing [44] (extrahepatic bile duct cancer and ampulla of vater cancer), Xiao [47], He [52], Chang [53], Cheng [57], Hsu [59] and Cai [62] were only calculated for the heterozygous model, dominant model and allele comparison for the *IL-10* -1082A/G polymorphism, and the number of GG genotype was zero.

^b^ Bai [44], Hao [46] and Niu [50] were only calculated for the dominant model.

For the studies assessing three polymorphisms (-1082A/G, -819T/C and -592A/C) [32, 37], two (-1082A/G and -592A/C) [31], only one such as -1082A/G [29, 30, 33-35, 38] or -819T/C [36, 39] polymorphism and cancer risk but no other *IL-10* gene polymorphisms, the genotypes distribution in the controls were deviated from HWE, thus, these studies were excluded in the final analysis. Sixteen studies were also deviated from HWE, but the genotypes distribution in the controls of eight studies [18, 64-67, 70, 73, 76] were consistent with that expected from the HWE for both -819T/C and -592A/C polymorphisms, five [81, 84, 86, 87, 90] for the -592A/C polymorphism and three [41, 48, 54] for the -1082A/G polymorphism, thus, these studies were included in the final analysis. For those studies [18, 45, 65] with the same control subjects, the control numbers were calculated once in the total number. Overall, 26 studies with 4,901 cases and 6,426 controls for the -1082A/G polymorphism, 33 studies with 6,717 cases and 8,550 controls for the -819T/C polymorphism, and 42 studies with 9,934 cases and 13,169 controls for the -592A/C polymorphism were considered in this meta-analysis. Sample sizes for cases of the included studies ranged from 43 to 400 for the -1082A/G polymorphism, 43 to 589 for the -819T/C polymorphism, and 70 to 720 for the -592A/C polymorphism.

As regards the -1082A/G polymorphism, five studies focused on gastric cancer [40, 44, 47, 52, 56], three on hepatocellular carcinoma [41, 49, 63], two studies for each of the following cancer types, such as lung cancer [42, 46], cervical cancer [51, 61] and esophageal cancer [55, 60], and the other cancer types with one study per each cancer type. As regards the -819T/C polymorphism, 10 studies focused on gastric cancer [18, 47, 52, 64, 65, 68, 70, 72, 73, 77], four on hepatocellular carcinoma [18, 49, 63, 69], three on esophageal cancer [18, 60, 65], two studies for each of the following cancer types, such as lung cancer [42, 75], oral cancer [59, 66] and acute myeloid leukemia [58, 74], and the other cancer types with one study per each cancer type. As regards the -592A/C polymorphism, 11 studies focused on gastric cancer [18, 47, 52, 56, 64, 65, 70, 73, 87, 89, 91], five on hepatocellular carcinoma [18, 63, 69, 78, 85], four studies for each of the following cancer types, such as lung cancer [42, 75, 80, 86] and esophageal cancer [18, 60, 65, 83], three on cervical cancer [61, 79, 81], two studies for each of the following cancer types, such as nasopharyngeal carcinoma [43, 84], oral cancer [59, 66], acute myeloid leukemia [58, 74] and colorectal cancer [62, 88], and the other cancer types with one study per each cancer type. Among all studies, 18 were hospital-based and eight were population-based associated to the -1082A/G polymorphism, 23 were hospital-based and 10 were population-based associated to the -819T/C polymorphism, 31 were hospital-based and 11 were population-based associated to the -592A/C polymorphism. Furthermore, 18 studies were rated as low quality (quality score ≤ 9) and eight were considered as high quality (quality score > 9) for the -1082A/G polymorphism, 16 were low quality and 17 were high quality studies for the -819T/C polymorphism, 21 were low quality and 21 were high quality studies for the -592A/C polymorphism. Controls were matched for age and sex in most studies, and cases were mostly histologically confirmed.

### Meta-analysis results

**Figure 2:**
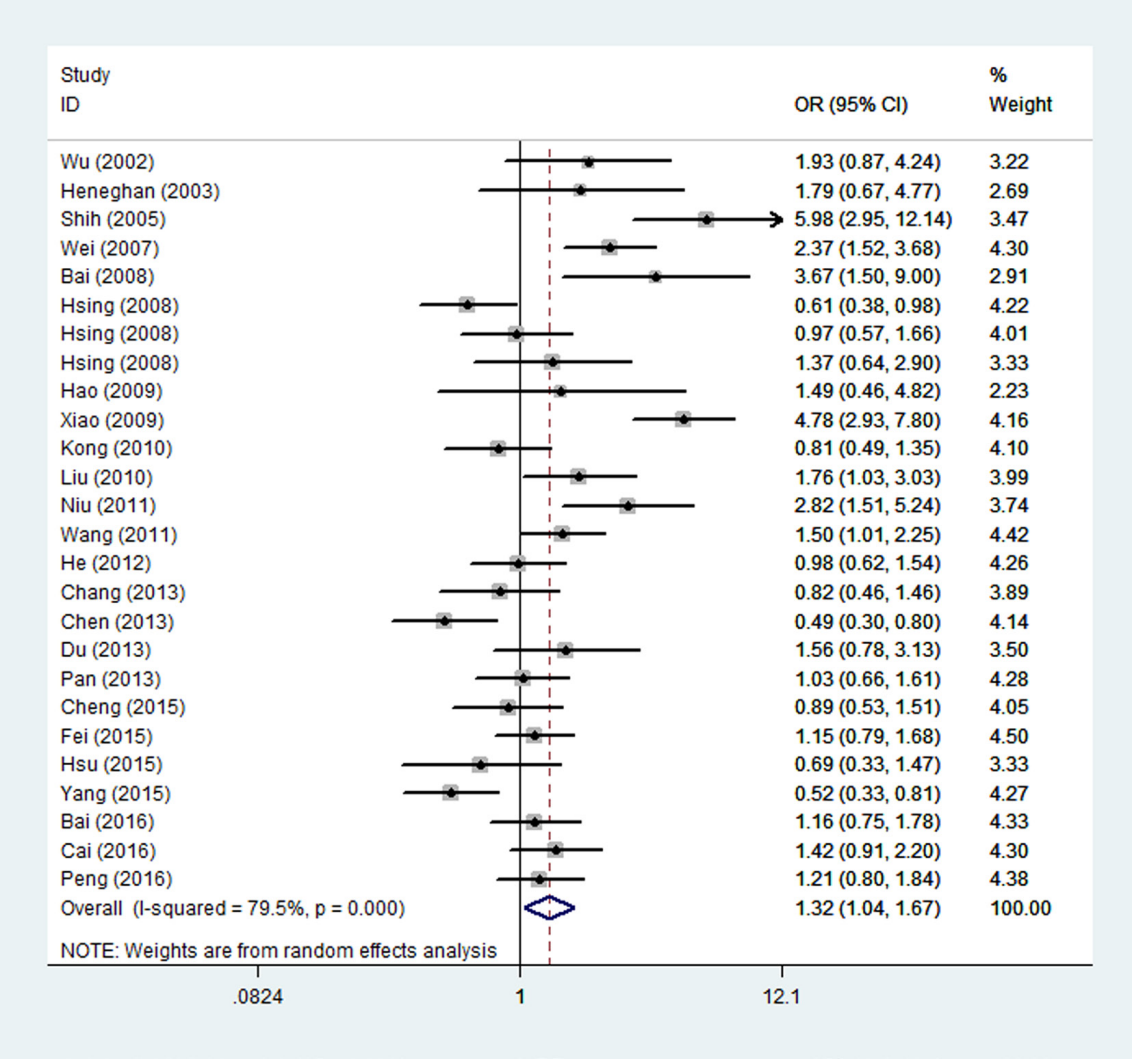
Forest plot for overall cancer risk associated with the IL-10 -1082A/G polymorphism by a dominant model For each study, the estimated OR and its 95% CI are plotted with a box and a horizontal line. ◊, pooled ORs and its 95% CIs.

The main results regarding the association between *IL-10* -1082A/G polymorphism and cancer risk are shown in Table 2 and Figure 2. A significant association was found between *IL-10* -1082A/G polymorphism and overall cancer risk [dominant: odds ratio (OR) = 1.32, 95% confidence interval (CI) = 1.04-1.67, *P* < 0.001]. In the subgroup analysis, a statistically significant association was found for hepatocellular carcinoma (heterozygous: OR = 1.40, 95% CI = 1.01-1.94, *P* = 0.433; dominant: OR = 1.43, 95% CI = 1.04-1.95, *P* = 0.497 and allele comparison: OR = 1.35, 95% CI = 1.04-1.75, *P* = 0.480) and low quality studies (heterozygous: OR = 1.42, 95% CI = 1.05-1.91, *P* < 0.001; dominant: OR = 1.56, 95% CI = 1.17-2.08, *P* < 0.001 and allele comparison: OR = 1.43, 95% CI = 1.08-1.88, *P* < 0.001).

**Table 2:** Meta-analysis of the association between IL-10 polymorphisms and cancer risk

Variables	No. of studies	Sample size (case/controls)	Homozygous		Heterozygous		Recessive		Dominant		Allele comparison	
			OR (95% CI)	*P*^het^	OR (95% CI)	*P*^het^	OR (95% CI)	*P*^het^	OR (95% CI)	*P*^het^	OR (95% CI)	*P*^het^
-1082A/G			GG *vs.* AA		AG *vs.* AA		GG *vs.*(AA+AG)		(AG+GG) *vs.* AA		G *vs.*A	
All	26	4,901/6,426	1.21 (0.80-1.85)	0.025	1.22 (0.97-1.54)	<0.001	1.12 (0.84-1.48)	0.242	**1.32 (1.04-1.67)**	<0.001	1.22 (0.99-1.51)	<0.001
Cancer type												
Gastric	5	985/1,511	1.38 (0.37-5.20)	0.930	1.70 (0.79-3.66)	<0.001	1.37 (0.36-5.13)	0.953	1.97 (0.97-3.99)	<0.001	1.72 (0.79-3.71)	<0.001
HCC	3	441/466	1.56 (0.77-3.18)	0.950	**1.40 (1.01-1.94)**	0.433	1.45 (0.73-2.90)	0.978	**1.43 (1.04-1.95)**	0.497	**1.35 (1.04-1.75)**	0.480
Lung ^a^	2	197/257	NA	NA	NA	NA	NA	NA	3.24 (0.84-12.54)	0.047	NA	NA
Cervical	2	351/365	1.45 (0.87-2.40)	0.792	1.31 (0.96-1.79)	0.371	1.26 (0.78-2.04)	0.991	1.33 (0.99-1.79)	0.386	1.24 (0.99-1.55)	0.490
Esophageal	2	364/611	0.88 (0.14-5.40)	0.099	0.88 (0.36-2.14)	0.041	0.94 (0.29-3.01)	0.205	0.87 (0.29-2.56)	0.009	1.00 (0.44-2.26)	0.015
Others	12	2,563/3,216	1.30 (0.59-2.85)	0.168	0.96 (0.74-1.25)	0.002	1.30 (0.68-2.46)	0.280	1.05 (0.78-1.41)	<0.001	0.97 (0.74-1.27)	<0.001
Source of control												
PB	8	1,025/1,398	1.42 (0.87-2.33)	0.454	1.13 (0.84-1.53)	0.114	1.25 (0.78-2.01)	0.538	1.29 (0.92-1.80)	0.013	1.07 (0.82-1.41)	0.078
HB	18	3,876/5,028	1.20 (0.69-2.09)	0.018	1.25 (0.93-1.68)	<0.001	1.13 (0.78-1.64)	0.183	1.33 (0.98-1.80)	<0.001	1.27 (0.97-1.68)	<0.001
Score												
Low	18	3,365/4,373	1.29 (0.78-2.12)	0.012	**1.42 (1.05-1.91)**	<0.001	1.16 (0.83-1.63)	0.160	**1.56 (1.17-2.08)**	<0.001	**1.43 (1.08-1.88)**	<0.001
High	8	1,536/2,053	1.13 (0.52-2.48)	0.349	0.89 (0.68-1.17)	0.073	1.15 (0.57-2.31)	0.417	0.88 (0.67-1.67)	0.059	0.88 (0.68-1.14)	0.047
-819T/C			CC *vs.*TT		CT *vs.*TT		CC *vs.*(TT+CT)		(CT+CC) *vs.*TT		C *vs.*T	
All	33	6,717/8,550	**1.19 (1.00-1.41)**	<0.001	1.04 (0.93-1.16)	<0.001	**1.17 (1.00-1.36)**	<0.001	1.08 (0.97-1.20)	<0.001	**1.08 (1.00-1.18)**	<0.001
Cancer type												
Gastric	10	1,772/2,142	1.08 (0.79-1.47)	0.021	1.15 (0.95-1.38)	0.046	1.01 (0.81-1.27)	0.196	1.14 (0.93-1.40)	0.007	1.08 (0.92-1.27)	0.002
HCC	4	1,118/1,344	1.14 (0.86-1.51)	0.744	0.96 (0.78-1.19)	0.396	1.04 (0.86-1.26)	0.668	1.00 (0.82-1.22)	0.412	1.01 (0.90-1.15)	0.549
Esophageal	3	558/873	1.23 (0.90-1.67)	0.940	1.02 (0.82-1.27)	0.741	1.21 (0.91-1.61)	0.966	1.07 (0.87-1.31)	0.763	1.09 (0.94-1.27)	0.852
Lung	2	484/541	**2.66 (1.84-3.84)**	0.569	1.18 (0.90-1.56)	0.560	**2.40 (1.71-3.37)**	0.399	**1.49 (1.16-1.92)**	0.633	**1.59 (1.33-1.91)**	0.920
Oral	2	425/412	**1.58 (1.01-2.46)**	0.464	1.77 (0.58-5.37)	0.001	1.35 (0.89-2.06)	0.583	1.80 (0.67-4.82)	0.002	1.38 (0.94-2.02)	0.080
AML	2	282/465	0.87 (0.22-3.48)	0.006	0.80 (0.32-2.01)	0.007	0.98 (0.38-2.53)	0.046	0.82 (0.29-2.34)	0.001	0.88 (0.38-2.03)	<0.001
Others	10	2,078/2,773	1.08 (0.76-1.53)	<0.001	0.91 (0.76-1.09)	0.047	1.14 (0.79-1.65)	<0.001	0.95 (0.82-1.11)	0.117	1.10 (0.87-1.18)	0.001
Source of control												
PB	10	1,502/1,872	1.24 (0.93-1.65)	0.035	0.96 (0.79-1.16)	0.035	1.31 (0.92-1.86)	<0.001	1.01 (0.88-1.16)	0.248	1.08 (0.95-1.24)	0.031
HB	23	5,215/6,678	1.17 (0.94-1.44)	<0.001	1.08 (0.95-1.22)	0.001	1.12 (0.95-1.33)	<0.001	1.10 (0.96-1.27)	<0.001	1.08 (0.97-1.20)	<0.001
Score												
Low	16	3,039/4,160	1.21 (0.89-1.64)	<0.001	1.07 (0.89-1.29)	<0.001	1.18 (0.92-1.51)	<0.001	1.11 (0.91-1.36)	<0.001	1.10 (0.94-1.29)	<0.001
High	17	3,678/4,390	1.17 (0.98-1.39)	0.075	1.01 (0.89-1.13)	0.097	1.16 (0.95-1.42)	0.001	1.03 (0.94-1.12)	0.409	1.05 (0.97-1.14)	0.089
-592A/C			CC *vs.*AA		AC *vs.*AA		CC *vs.*(AA+AC)		(AC+CC) *vs.*AA		C *vs.*A	
All	42	9,934/13,169	**1.13 (1.00-1.28)**	0.001	1.04 (0.96-1.13)	0.001	1.10 (0.99-1.21)	0.035	1.06 (0.97-1.15)	<0.001	1.05 (0.99-1.12)	<0.001
Cancer type												
Gastric	11	2,324/2,775	1.18 (0.96-1.44)	0.289	1.08 (0.94-1.23)	0.200	1.11 (0.94-1.32)	0.562	1.10 (0.95-1.27)	0.093	1.08 (0.97-1.21)	0.080
HCC	5	1,859/2,109	1.20 (0.82-1.75)	0.032	1.09 (0.94-1.27)	0.650	1.10 (0.80-1.50)	0.039	1.09 (0.94-1.27)	0.373	1.08 (0.93-1.24)	0.094
Esophageal	4	900/1,243	1.18 (0.90-1.54)	0.637	1.13 (0.93-1.36)	0.399	1.11 (0.88-1.39)	0.498	1.15 (0.96-1.37)	0.582	1.10 (0.97-1.25)	0.702
Lung	4	958/1,377	**1.64 (1.19-2.24)**	0.301	1.17 (0.94-1.45)	0.285	**1.52 (1.20-1.93)**	0.402	**1.27 (1.01-1.60)**	0.198	**1.27 (1.06-1.52)**	0.149
Cervical	3	338/388	0.89 (0.35-2.24)	0.031	0.91 (0.67-1.25)	0.431	0.94 (0.41-2.19)	0.042	0.89 (0.60-1.32)	0.174	0.91 (0.60-1.38)	0.034
NPC	2	374/732	1.19 (0.62-2.31)	0.116	0.95 (0.72-1.25)	0.697	1.22 (0.66-2.25)	0.125	0.99 (0.77-1.29)	0.346	1.05 (0.78-1.42)	0.129
Oral	2	425/412	**1.58 (1.01-2.46)**	0.464	1.77 (0.58-5.37)	0.001	1.35 (0.89-2.06)	0.583	1.80 (0.67-4.82)	0.002	1.38 (0.94-2.02)	0.080
AML	2	282/465	0.84 (0.23-3.05)	0.011	0.79 (0.33-1.90)	0.010	0.95 (0.40-2.27)	0.064	0.80 (0.30-2.16)	0.002	0.86 (0.39-1.88)	0.001
Colorectal	2	673/673	**0.58 (0.40-0.85)**	0.694	**0.66 (0.53-0.83)**	0.882	0.70 (0.49-1.01)	0.599	**0.65 (0.52-0.80)**	0.994	**0.72 (0.61-0.85)**	0.750
Others	7	1,801/2,995	0.98 (0.77-1.24)	0.313	1.01 (0.86-1.17)	0.246	0.98 (0.80-1.21)	0.437	1.00 (0.86-1.16)	0.185	0.99 (0.88-1.11)	0.187
Source of control												
PB	11	2,203/2,780	1.08 (0.82-1.43)	0.011	0.96 (0.82-1.13)	0.056	1.08 (0.89-1.33)	0.117	0.99 (0.82-1.18)	0.004	1.01 (0.87-1.16)	0.001
HB	31	7,731/10,389	1.14 (0.99-1.31)	0.009	1.07 (0.97-1.17)	0.004	1.10 (0.98-1.24)	0.054	1.09 (0.99-1.20)	<0.001	**1.07 (1.00-1.15)**	<0.001
Score												
Low	21	4,240/6,041	**1.23 (1.02-1.49)**	0.012	1.03 (0.90-1.19)	<0.001	**1.21 (1.05-1.40)**	0.193	1.08 (0.93-1.25)	<0.001	1.09 (0.98-1.21)	<0.001
High	21	5,694/7,128	1.05 (0.89-1.23)	0.023	1.05 (0.96-1.15)	0.161	1.02 (0.89-1.16)	0.100	1.05 (0.95-1.15)	0.033	1.03 (0.95-1.11)	0.007

Het: heterogeneity; NA: not applicable; HCC: hepatocellular carcinoma; NPC: nasopharyngeal carcinoma; AML: acute myeloid leukemia; PB: population based; HB: hospital based.

^a^ Lung cancer was only calculated for the dominant model.

The overall results regarding the association between *IL-10* -819T/C polymorphism and cancer risk are shown in Table 2. A significant association was found between *IL-10* -819T/C polymorphism and overall cancer risk (homozygous: OR = 1.19, 95% CI = 1.00-1.41, *P* < 0.001; recessive: OR = 1.17, 95% CI = 1.00-1.36, *P* < 0.001 and allele comparison: OR = 1.08, 95% CI = 1.00-1.18, *P* < 0.001). In the subgroup analysis, a statistically significant association was found for lung cancer (homozygous: OR = 2.66, 95% CI = 1.84-3.84, *P* = 0.569; recessive: OR = 2.40, 95% CI = 1.71-3.37, *P* = 0.399; dominant: OR = 1.49, 95% CI = 1.16-1.92, *P* = 0.633 and allele comparison: OR = 1.59, 95% CI = 1.33-1.91, *P* = 0.920) and oral cancer (homozygous: OR = 1.58, 95% CI = 1.01-2.46, *P* = 0.464).

The detailed results regarding the association between *IL-10* -592A/C polymorphism and cancer risk are shown in Table 2. A significant association was found between *IL-10* -592A/C polymorphism and increased overall cancer risk (homozygous: OR = 1.13, 95% CI = 1.00-1.28, *P* = 0.001). In the subgroup analysis, a statistically significant increased risk was found for lung cancer (homozygous: OR = 1.64, 95% CI = 1.19-2.24, *P* = 0.301; recessive: OR = 1.52, 95% CI = 1.20-1.93, *P* = 0.402; dominant: OR = 1.27, 95% CI = 1.01-1.60, *P* = 0.198 and allele comparison: OR = 1.27, 95% CI = 1.06-1.52, *P* = 0.149), oral cancer (homozygous: OR = 1.58, 95% CI = 1.01-2.46, *P* = 0.464), hospital-based studies (allele comparison: OR = 1.07, 95% CI = 1.00-1.15, *P* < 0.001) and low quality studies (homozygous: OR = 1.23, 95% CI = 1.02-1.49, *P* = 0.012 and recessive: OR = 1.21, 95% CI = 1.05-1.40, *P* = 0.193). In contrast, a significantly decreased risk was observed for colorectal cancer (homozygous: OR = 0.58, 95% CI = 0.40-0.85, *P* = 0.694; heterozygous: OR = 0.66, 95% CI = 0.53-0.83, *P* = 0.882; dominant: OR = 0.65, 95% CI = 0.52-0.80, *P* = 0.994 and allele comparison: OR = 0.72, 95% CI = 0.61-0.85, *P* = 0.750).

### Heterogeneity and sensitivity analysis

Substantial heterogeneities were found among all studies regarding *IL-10* -1082A/G polymorphism and overall cancer risk (homozygous: *P* = 0.025; heterozygous: *P* < 0.001; dominant: *P* < 0.001 and allele comparison: *P* < 0.001), but not under the recessive model (*P* = 0.242) (Table 2). Considerable heterogeneities were also observed for the -819T/C (all *P* < 0.001) and -592A/C (homozygous: *P* = 0.001; heterozygous: *P* = 0.001; recessive: *P* = 0.035; dominant: *P* < 0.001 and allele comparison: *P* < 0.001) polymorphisms. Therefore, the random-effect model was used to generate wider CIs. Sensitivity analysis was conducted and the results indicated that each individual study did not influence the pooled ORs obviously (data not shown).

### Publication bias

**Figure 3:**
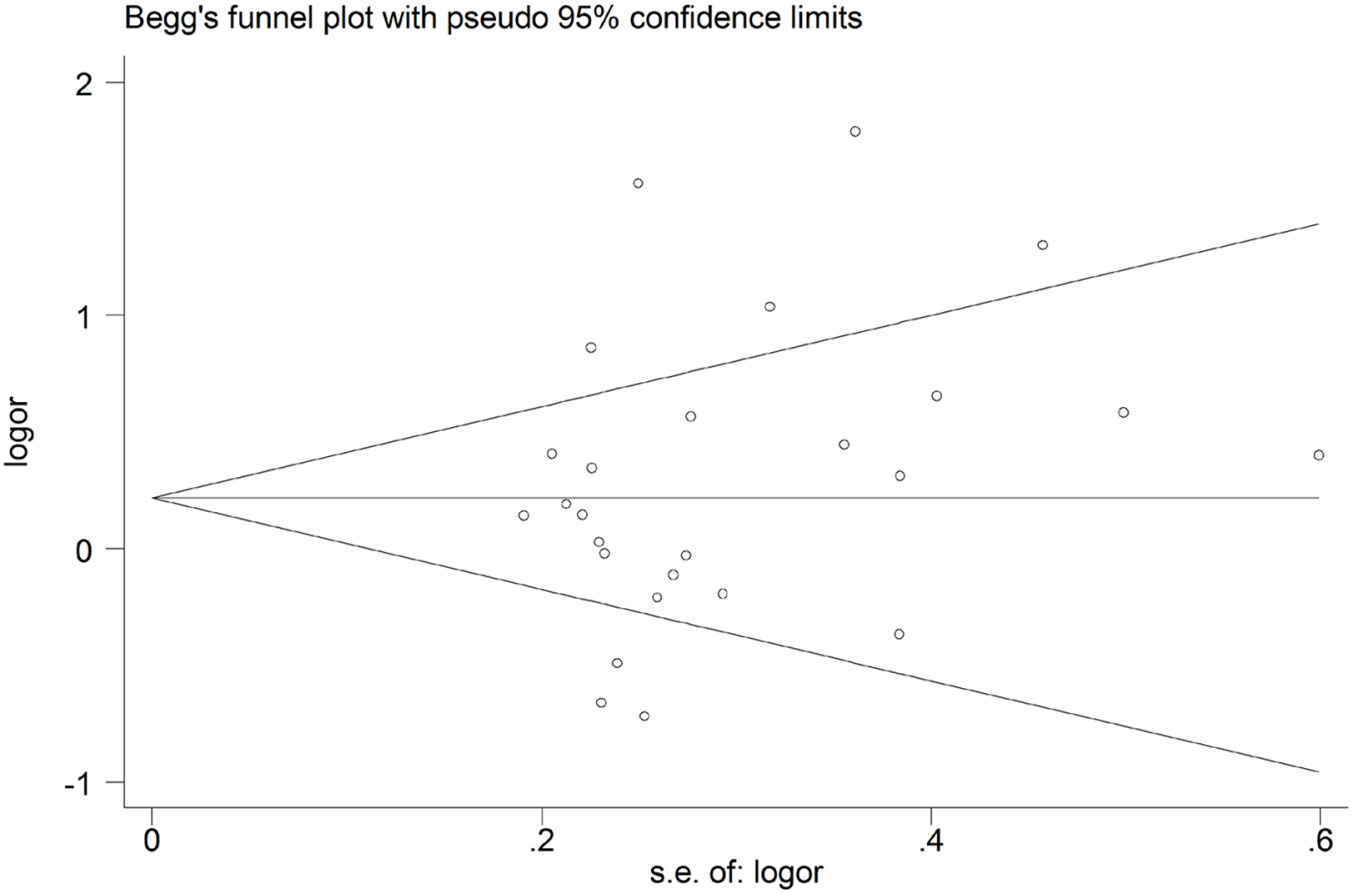
Begg’s funnel plot for the *IL-10* -1082A/G polymorphism and overall cancer risk by a dominant model

The funnel plot was symmetric for the -1082A/G (Figure 3), -819T/C and -592A/C polymorphisms, indicating no presence of publication bias, which was further supported by the Egger’s test for the -1082A/G polymorphism (homozygous: *P* = 0.428; heterozygous: *P* = 0.395; recessive: *P* = 0.168; dominant: *P* = 0.223 and allele comparison: *P* = 0.179), -819T/C polymorphism (homozygous: *P* = 0.589; heterozygous: *P* = 0.777; recessive: *P* = 0.616; dominant: *P* = 0.797 and allele comparison: *P* = 0.576), and -592A/C polymorphism (homozygous: *P* = 0.727; heterozygous: *P* = 0.763; recessive: *P* = 0.748; dominant: *P* = 0.474 and allele comparison: *P* = 0.677).

### False-positive report probability (FPRP) test analysis

The significant findings were assessed using the FPRP test and the results are shown in Table 3. With a prior probability of 0.1, assuming that the OR for a specific genotype was 0.67/1.50 (protection/risk), with statistical power of 0.857, the FPRP value was 0.179 for the -1082A/G polymorphism and cancer risk under the dominant model, and a positive association was also found for low quality studies (dominant: FPRP = 0.053 and allele comparison: FPRP = 0.129). As regards the -819T/C polymorphism, a positive association was found for lung cancer (homozygous: FPRP = 0.001; recessive: FPRP = 0.001; dominant: FPRP = 0.034 and allele comparison: FPRP < 0.001). As regards the -592A/C polymorphism, noteworthy findings were observed for lung cancer (homozygous: FPRP = 0.055; recessive: FPRP = 0.011 and allele comparison: FPRP = 0.078), colorectal cancer (homozygous: FPRP = 0.165; heterozygous: FPRP = 0.007; dominant: FPRP = 0.001 and allele comparison: FPRP = 0.001) and low quality studies (recessive: FPRP = 0.086). However, greater FPRP values were observed for other significant findings, which need validation in further studies.

**Table 3:** False-positive report probability values for associations between cancer risk and *IL-10* polymorphisms

Genotype	Crude OR (95% CI)	*P*-valuea	Statistical powerb	Prior probability				
				0.25	0.1	0.01	0.001	0.0001
-1082A/G								
All								
Dominant	1.32 (1.04-1.67)	0.021	0.857	**0.068**	**0.179**	0.705	0.960	0.996
Cancer type-HCC								
Heterozygous	1.40 (1.01-1.94)	0.043	0.661	**0.164**	0.371	0.866	0.985	0.998
Dominant	1.43 (1.04-1.95)	0.024	0.619	**0.103**	0.257	0.792	0.975	0.997
Allele comparison	1.35 (1.04-1.75)	0.023	0.787	**0.082**	0.211	0.747	0.967	0.997
Quality score-low								
Heterozygous	1.42 (1.05-1.91)	0.020	0.641	**0.087**	0.223	0.759	0.970	0.997
Dominant	1.56 (1.17-2.08)	0.002	0.395	**0.018**	**0.053**	0.380	0.861	0.984
Allele comparison	1.43 (1.08-1.88)	0.010	0.634	**0.047**	**0.129**	0.619	0.942	0.994
-819T/C								
All								
Homozygous	1.19 (1.00-1.41)	0.044	0.996	**0.118**	0.286	0.815	0.978	0.998
Recessive	1.17 (1.00-1.36)	0.041	0.999	**0.109**	0.269	0.802	0.976	0.998
Allele comparison	1.08 (1.00-1.18)	0.088	1.000	0.210	0.443	0.898	0.989	0.999
Cancer type-lung cancer								
Homozygous	2.66 (1.84-3.84)	<0.001	0.001	**<0.001**	**0.001**	**0.015**	**0.137**	0.613
Recessive	2.40 (1.71-3.37)	<0.001	0.003	**<0.001**	**0.001**	**0.013**	**0.114**	0.564
Dominant	1.49 (1.16-1.92)	0.002	0.521	**0.012**	**0.034**	0.281	0.797	0.975
Allele comparison	1.59 (1.33-1.91)	<0.001	0.267	**<0.001**	**<0.001**	**<0.001**	**0.003**	**0.026**
Cancer type-oral cancer								
Homozygous	1.58 (1.01-2.46)	0.043	0.409	0.239	0.485	0.912	0.991	0.999
-592A/C								
All								
Homozygous	1.13 (1.00-1.28)	0.055	1.000	**0.141**	0.330	0.844	0.982	0.998
Cancer type-lung cancer								
Homozygous	1.64 (1.19-2.24)	0.002	0.287	**0.019**	**0.055**	0.392	0.867	0.985
Recessive	1.52 (1.20-1.93)	0.001	0.457	**0.004**	**0.011**	**0.113**	0.563	0.928
Dominant	1.27 (1.01-1.60)	0.043	0.921	**0.122**	0.294	0.821	0.979	0.998
Allele comparison	1.27 (1.06-1.52)	0.009	0.965	**0.028**	**0.078**	0.484	0.904	0.990
Cancer type-oral cancer								
Homozygous	1.58 (1.01-2.46)	0.043	0.409	0.239	0.485	0.912	0.991	0.999
Cancer type-colorectal cancer								
Homozygous	0.58 (0.40-0.85)	0.005	0.238	**0.062**	**0.165**	0.685	0.956	0.995
Heterozygous	0.66 (0.53-0.83)	<0.001	0.466	**0.002**	**0.007**	**0.075**	0.449	0.891
Dominant	0.65 (0.52-0.80)	<0.001	0.406	**<0.001**	**0.001**	**0.012**	0.105	0.541
Allele comparison	0.72 (0.61-0.85)	<0.001	0.818	**<0.001**	**0.001**	**0.013**	0.113	0.562
Control source-HB								
Allele comparison	1.07 (1.00-1.15)	0.066	1.000	**0.165**	0.372	0.867	0.985	0.998
Quality score-low								
Homozygous	1.23 (1.02-1.49)	0.034	0.979	**0.095**	0.240	0.777	0.972	0.997
Recessive	1.21 (1.05-1.40)	0.010	0.998	**0.030**	**0.086**	0.508	0.913	0.991

HCC: hepatocellular carcinoma; HB: hospital based.

^a^Chi-square test was used to calculate the genotype frequency distributions.

^b^Statistical power was calculated using the number of observations in the subgroup and the OR and *P* values in this table.

## DISCUSSION

In this meta-analysis, we comprehensively investigated the associations between three promoter variants (-1082A/G, -819T/C and -592A/C) in *IL-10* gene and cancer risk in the Chinese population through 53 articles. The results revealed that all the three *IL-10* gene polymorphisms we considered were associated with an increased overall cancer risk. Stratification analysis showed that the association between the -1082A/G polymorphism and cancer risk was more evident for hepatocellular carcinoma and low quality studies, the association between the -819T/C polymorphism and cancer risk was more obvious for lung cancer and oral cancer. However, the -592A/C polymorphism showed a statistically significant increased risk for lung cancer, oral cancer, hospital-based studies and low quality studies, but a decreased risk for colorectal cancer. To our knowledge, this is so far the first meta-analysis that has assessed multiple promoter polymorphisms in *IL-10* gene with cancer risk in the Chinese population.

Three meta-analyses including international studies have investigated the association of *IL-10* -1082A/G, -819T/C and -592A/C polymorphisms with overall cancer susceptibility. The study carried out by Wang *et al.* [92] analyzed *IL-10* -1082A/G polymorphism, consisting 61 international studies with a total of 14,499 cases and 16,967 controls, in which no significant association was found between this polymorphism and overall cancer risk. Another meta-analysis [93] including 15,942 cases and 22,336 controls investigated *IL-10* -819C/T polymorphism and cancer risk, without finding any significant association between this polymorphism and overall cancer risk. The study carried out by Ding *et al.* [94] considered *IL-10* -592C/A polymorphism, in which a decreased risk of overall cancer was found with the AA genotype. Other meta-analyses with international studies have assessed the association between polymorphisms in *IL-10* gene and susceptibility to some types of cancer. For example, two studies [95, 96] revealed no significant association between *IL-10* -1082A/G, -819T/C and -592A/C polymorphisms with non-Hodgkin lymphoma susceptibility. Some of the significant associations found in the previous studies were not validated in our meta-analysis, for example, *IL-10* -1082A/G polymorphism was associated with an increased lung cancer risk [92]. We also found some significant associations that were not observed in previous analyses. For instance, we found that *IL-10* -592A/C polymorphism was associated with a decreased colorectal cancer risk. The discrepancy occurred because our analysis was carried out only in the Chinese population, suggesting that the polymorphisms on cancer risk might vary among different study subjects’ ethnicity or lifestyle factors.

To make our significant findings more noteworthy, FPRP analysis was performed. Interestingly, FPRP test results revealed that only the association between *IL-10* -1082A/G polymorphism and overall cancer risk remained significant at the prior probability level of 0.1. In the subgroup analysis, only the low quality studies, lung cancer and colorectal cancer remained significant. Other findings were false-positive, which might be due to the limited sample size.

Our present meta-analysis has some highlights. First, it identified the significant association between *IL-10* -1082A/G, -819T/C and -592A/C polymorphisms and an increased overall cancer risk in the Chinese population. Second, the quality of each included study was evaluated by the quality score criteria. Third, no publication bias was detected in the study, indicating the robustness of the results. Finally, the significant findings were further validated using the FPRP test, making the results more authentic. However, some possible limitations should be considered. First, the total sample size in each individual study was less than 1000 in all but four studies [69, 82, 85, 86], which might reflect a difficulty to evaluate the real association. Second, our results were based on unadjusted estimates, which might cause confounding bias. Third, in the subgroup analysis by cancer type, only two studies were included for some types of cancer, which might affect the detection of the real association. Finally, the potential gene-gene, and gene-environment interactions were not assessed due to the lack of information in the original studies.

In conclusion, this meta-analysis suggested an association between *IL-10* gene polymorphisms and cancer risk in the Chinese population, especially for lung cancer, colorectal cancer and low quality studies. Well-designed studies with large sample size are required to verify our findings.

## MATERIALS AND METHODS

### Search strategy

A systematic literature search was conducted in PubMed, Embase, CNKI and Wanfang database using the following MeSH terms and their synonyms: (“*interleukin-10*” or “*interleukin 10*” or “*IL-10*” or “*IL 10*”) AND (“polymorphism, single nucleotide” [MeSH] or “SNP” or “single nucleotide polymorphism” or “polymorphism” or “variant” or “variation”) AND (“neoplasms” [MeSH] or “neoplasia” or “neoplasm” or “tumor” or “malignancy” or “cancer”), up to 19 January, 2017. In addition, review articles and references of the selected articles were manually searched to identify additional relevant articles. Only the most recent publications or the ones with most participants were included in the final meta-analysis in cases of overlapping data.

### Inclusion and exclusion criteria

The inclusion criteria were as follows: (1) studies investigating the association between *IL-10* -1082A/G, -819T/C and -592A/C polymorphisms with cancer risk in Chinese populations; (2) case-control studies; (3) studies providing sufficient data for calculation of ORs and 95% CIs. Studies were excluded if any of the following aspects existed: (1) not a case-control study; (2) duplicate publications; (3) studies without available genotype data; (4) review articles, meta-analyses, conference abstracts or editorial articles; and (5) genotype frequencies in the controls departure from HWE.

### Data extraction

Two investigators independently extracted the relevant data from all included studies based on the inclusion criteria listed above. Disagreement was resolved by discussion with a third investigator. The following information was extracted from each included study: first author’s surname, publication year, cancer type, control source (hospital-based or population-based), genotyping methods, and number of cases and controls with different genotypes.

### Quality assessment

Two independent investigators assessed the qualities of all included studies according to the criteria from a previous meta-analysis [97]. Quality scores of studies ranged from 0 (lowest) to 15 (highest), and the studies with scores > 9 were considered of high quality.

### Statistical analysis

The strength of association between *IL-10* -1082A/G, -819T/C and -592A/C polymorphisms and cancer risk was assessed by calculating the ORs and the corresponding 95% CIs. The pooled ORs were calculated for the homozygous model, heterozygous model, recessive model, dominant model and an allele comparison. The between-study heterogeneity was quantified by chi-square based Q test and the fixed-effects model (the Mantel-Haenszel method) [98] was used when no significant heterogeneity was observed (*P* > 0.1); otherwise, the random-effects model (the DerSimonian and Laird method) [99] was adopted. Subgroup analysis was performed by cancer type (if one cancer type contained less than two studies, it was merged into the “other cancers” group), control source (hospital-based studies and population-based studies), and quality scores (≤ 9 and > 9). Sensitivity analysis was performed to assess results stability. Publication bias was examined using Begg’s funnel plot and Egger’s linear regression test.

The FPRP was calculated to examine the significant associations found in the present meta-analysis. FPRP was calculated with 0.2 as a FPRP threshold and a prior probability of 0.1 was assigned to detect an OR of 0.67/1.50 (protective/risk effects) for an association with the genotypes under investigation [100]. FPRP values below threshold 0.2 were considered as noteworthy associations. All the statistical tests were performed using STATA version 12.0 (Stata Corporation, College Station, TX). All the *P* values were two-sided, and *P* < 0.05 were considered statistically significant.
